# The Impact, Emerging Needs, and New Research Questions Arising from 12 Years of the Center for the Study of Complex Malaria in India

**DOI:** 10.4269/ajtmh.21-1277

**Published:** 2022-10-13

**Authors:** Jane M. Carlton, Praveen K. Sahu, Samuel C. Wassmer, Sanjib Mohanty, Anne Kessler, Alex Eapen, Sheena Shah Tomko, Catherine Walton, Pyare L. Joshi, Deben Das, Sandra Albert, Bennichan K. Peter, Madan M. Pradhan, Aditya P. Dash, Aparup Das

**Affiliations:** ^1^ Department of Biology, Center for Genomics and Systems Biology, New York University, New York, New York;; ^2^ Department of Epidemiology, School of Global Public Health, New York University, New York, New York;; ^3^ Department of Molecular and Infectious Diseases, Community Welfare Society Hospital, Rourkela, India;; ^4^ Department of Infection Biology, London School of Hygiene and Tropical Medicine, London, United Kingdom;; ^5^ Department of Molecular and Infectious Diseases, Community Welfare Society Hospital, Rourkela, India;; ^6^ IDVC Field Unit, National Institute of Malaria Research, Indian Council of Medical Research, National Institute of Epidemiology Campus, Chennai, India;; ^7^ Department of Biology, University of Pennsylvania, Philadelphia, Pennsylvania;; ^8^ Department of Earth and Environmental Sciences, School of Natural Sciences, University of Manchester, Manchester, United Kingdom;; ^9^ Joint Scientific Advisory Committee, Indian Council of Medical Research, and Malaria No More, India Programme, New Delhi, India;; ^10^ District Headquarters Hospital, Keonjhar, India;; ^11^ Indian Institute of Public Health—Shillong, Shillong, India;; ^12^ Martin Luther Christian University, Shillong, India;; ^13^ Community Welfare Society Hospital, Rourkela, India;; ^14^ Department of Health and Family Welfare, State Vector Borne Disease Control Programme, Bhubaneswar, India;; ^15^ Asian Institute of Public Health University, Bhubaneswar, India;; ^16^ National Institute of Research in Tribal Health, Indian Council of Medical Research, Jabalpur, India

## Abstract

The Center for the Study of Complex Malaria in India (CSCMi) was launched in 2010 with the overall goal of addressing major gaps in our understanding of “complex malaria” in India through projects on the epidemiology, transmission, and pathogenesis of the disease. The Center was mandated to adopt an integrated approach to malaria research, including building capacity, developing infrastructure, and nurturing future malaria leaders while conducting relevant and impactful studies to assist India as it moves from control to elimination. Here, we will outline some of the interactions and impacts the Center has had with malaria policy and control counterparts in India, as well as describe emerging needs and new research questions that have become apparent over the past 12 years.

## INTRODUCTION AND MALARIA CONTROL INITIATIVES IN INDIA

The Center for the Study of Complex Malaria in India (CSCMi), one of the 10 National Institutes of Health (NIH)-funded International Centers of Excellence in Malaria Research (ICEMR) located in malaria-endemic regions worldwide, was launched in 2010 with the overall goal of addressing major gaps in our understanding of “complex malaria” in India through research on the epidemiology, transmission, and pathogenesis of the disease.^1^ A summary of the Center’s work undertaken in six different regions (National Capital Territory of Delhi and five states: Odisha, Gujarat, Tamil Nadu, Madhya Pradesh, and Meghalaya) is provided in the accompanying manuscript^2^ (see also http://malariacenterindia.org). Although many of the projects involved basic science methods, the Center was mandated to adopt an integrated approach to malaria research,^3^ including building capacity, developing infrastructure, and nurturing future malaria leaders, as well as undertaking relevant and impactful research that would assist as India moves from control to elimination. Here, we will first describe Government of India (GOI) initiatives and institutes that have been developed for malaria control and elimination, followed by a description of interactions between the CSCMi and GOI malaria research, policy and control counterparts, and the impact that CSCMi research is having on malaria policy and public health. Finally, we will discuss the emerging needs and new research questions that have arisen as a result of our activities over the past 12 years.

Government of India malaria control initiatives date back to 1953, when the National Malaria Control Program (NMCP) was launched with a primary focus on vector control by indoor residual spraying (IRS) of DDT, and whose encouraging results spawned a more ambitious program, the National Malaria Eradication Program (NMEP), in 1958. Although the NMEP drastically reduced malaria-related deaths and case numbers, the development of mosquito resistance to insecticides and parasite resistance to antimalarial drugs led to malaria resurgence in the country. With this, the focus was shifted to “control” rather than eradication. The National Vector Borne Disease Control Program (NVBDCP, 2003) and the National Rural Health Mission (NRHM, 2005) have since instituted broad programs focusing on controlling several vector-borne diseases, including malaria. These operational aspects of malaria control are complemented by basic, applied and field research undertaken by the National Institute of Malaria Research (NIMR), a research institute of the Indian Council of Medical Research (ICMR), established in 1977. ICMR-NIMR has a network of research laboratories in Delhi and eight field stations in malarious areas that serve as a testing ground for new technologies and their implementation.

More recently, the National Framework for Malaria Elimination in India (2016–2030; http://www.indiaenvironmentportal.org.in/files/file/National-framework-for-malaria-elimination-in-India-2016–2030.pdf), developed in tandem with the World Health Organization Global Technical Strategy for Malaria (2016–2030), aims to eliminate malaria throughout the country by 2030. Another 5-year plan, the National Strategic Plan for Malaria Elimination, 2017–2022 (http://www.indiaenvironmentportal.org.in/files/file/nsp_2017-2022-updated.pdf) has also shifted focus from control to elimination, providing a roadmap to end malaria in a majority of districts in India by 2022, through strengthening surveillance and promoting the use of long lasting insecticidal nets (LLINs) and IRS.

## INTERACTIONS BETWEEN CSCMI AND MALARIA RESEARCH, POLICY AND CONTROL COUNTERPARTS, AND MALARIA COMMUNITIES IN INDIA

A large-scale, multiinvestigator, multiproject, and multidisciplinary international collaborative effort requires many levels of interaction among stakeholders at all levels. In the following, we outline some of the ways the CSCMi has achieved this.

### Engagement with Government of India malaria research and control programs.

A key approach throughout the development and operation of the CSCMi has been to continually engage with partners at GOI hospitals (e.g., Steel Authority of India Ltd., Ispat General Hospital [IGH] in Rourkela, Odisha), ICMR-NIMR headquarters in Delhi and its field stations, and the NVBDCP, to codevelop and prioritize the program of research. This has ensured that national needs were considered while framing the research questions so that the projects being undertaken are relevant, significant, and strategic, and provide opportunities for training, capacity building and transfer of new technology—an approach made easier by existing scientific collaborations between researchers at New York University and scientists at the ICMR-NIMR through a NIH/Fogarty International Center D43 International Research and Training Grant awarded in 2007 (Principal Investigators: Drs. A. P. Dash, Hema Joshi, and Jane Carlton). For example, during initial discussions with ICMR-NIMR prior to the Center being funded, the overall theme of studying the complexity of malaria was chosen, and field sites selected according to research themes: a rural field site with mixed *Plasmodium vivax* and *Plasmodium falciparum* (Nadiad in Gujarat state), a tribal field site where *P. falciparum* predominates (Rourkela in Odisha state), and an urban *P. vivax* field site (Chennai in Tamil Nadu state). After multiple meetings, research topics were identified including urban malaria, asymptomatic infections, transmission dynamics, insecticide resistance, antimalarial drug resistance, and so on. The 7-year length of the ICEMR program meant that epidemiological cohorts could be designed with follow-up over years. The ICEMR program was deemed by those involved in the initial ICMR-NIMR discussions (the NIMR Director, field officers, and scientists) to be a mutually beneficial collaboration, with a mix of different types of world class research to be carried out at the field level, and with much to gain and much to learn on both the endemic country and nonendemic country sides and, therefore, the collaboration was formalized. Throughout the Center’s operation there has been continuous real-time dissemination of research findings at the local as well as central level, for example, through presentations at the Conference of Vectors and Vector Borne Diseases held every 2 years in India by the National Academy of Vectors and Vector Borne Diseases.^4^

### Stakeholder meetings—the key to continuing strong partnerships.

Throughout the operation of the India ICEMR, we have held Stakeholder Engagement and Evidence Presentation (SEEP) events to inform major stakeholders of the Center’s overall goal, aims and progress, provide a forum for stakeholders to stay involved in operations, and provide guidance and assessment of progress for midstage study adjustments. The role of research in public health is not often obvious to policy stakeholders. Research teams may even be perceived by some in government as inconsequential, a hindrance, or even a “threat.” Against this SEEP events provide an opportunity to present work done, discuss field challenges, improve understanding on the purpose of the research, find potential solutions, forge partnerships, and develop new questions jointly.

Two examples of SEEP events for stakeholders in the Meghalaya site are illustrative. A stakeholder meeting was held in Guwahati, Assam, in September 2019. While appreciating the work done by the CSCMi, Government stakeholders reiterated the importance of initiating work in the Garo Hills, which had been deferred due to safety concerns; in response, we initiated work in this region in 2020. It was also noted to us that the malaria incidence pattern was changing to the “outbreaks” of *P. vivax* infections in the low-prevalence region of the Jaintia Hills. The CSCMi adapted and switched work to the Nartiang PHC area in Jaintia Hills, which had had little malaria in the prior 2 years but experienced an outbreak of vivax malaria in 2019. A stakeholder event held in November 2021 in Shillong, Meghalaya, featured a “show and tell” counter displaying all the instruments used in the field, to give government stakeholders an opportunity to get a firsthand feel for the type of field work being done. Challenges of working in difficult terrain, especially in the Garo Hills, were conveyed through images. The Meghalaya Government expressed appreciation for the hard work being done by the CSCMi, and posed several policy questions, including whether PCR-positive asymptomatic infections warrant treatment, and the role of molecular identification of vectors in the future.

### Engagement with malaria-impacted communities.

One of the most important interactions has been between CSCMi team members and malaria-impacted communities before, during, and at the conclusions of a study, to ensure community education, cooperation, and buy-in. As an example, interactions with village communities in Meghalaya are described here. Once all GOI approvals have been obtained, the CSCMi field teams meet with local health officials and clinicians at the Community Health Center (CHC) or Primary Health Center (PHC) to introduce themselves and describe the study aims and procedures. This has included both formal presentations and small group discussions to answer any questions that arise. Study team members then meet with village-level health workers, including accredited social health activists (ASHAs; local women trained to act as health educators and promoters in their communities) and Anganwadi (basic healthcare) workers, and with village headmen and the secretary, to obtain approval and consent for working in the villages. These introductions are key to being able to work in malaria-endemic communities in Meghalaya, and ensure that the very people who are impacted are kept informed and provided with opportunities for feedback that enhance the Center’s activities.

## IMPACT OF INDIAN ICEMR ON MALARIA POLICY AND PUBLIC HEALTH

Our continuous engagement with GOI malaria research institutes and hospitals has led to some of our research findings directly influencing malaria policy and public health in India.

### Malaria vector control in the urban setting of Chennai, Tamil Nadu.

Our studies on malaria vector behavior in Chennai identified rooftop water tanks as the predominant breeding habitat for the urban vector *Anopheles stephensi*^5^ and cattle sheds as its preferred place for both resting and feeding, and a preference for thatched structures among those found in human dwellings.^6^^,^^7^ We also found a strong positive correlation between the presence of fluoride and *An. stephensi* immature density in breeding habitats,^8^ as well as detected malaria parasites in *Anopheles subpictus*, which was not previously considered a human malaria vector in Chennai.^7^ Finally, we recently identified other factors such as roof type associated with the presence of *An. stephensi* in urban slums in Chennai.^9^ These findings led to policy changes by the NVBDCP in Chennai regarding new antilarval measures in breeding habitats. Some of our trained field team members are now coordinating vector control activities, for example, consulting in the GOI Regional Office for Health and Family Welfare at Besant Nagar, Chennai.

### Effectiveness studies team CSCMi researchers with the Malaria Control Program in Odisha.

A major study site of the CSCMi is in the highly endemic state of Odisha, whose 4% of the Indian population is historically responsible for 40% of malaria.^10^ In 2017, the Malaria Control Program (MCP) of the Odisha State Government rolled out a large-scale public health initiative called “Durgama Anchalare Malaria Nirakarana (DAMaN)” (“Malaria Elimination in Hard-to-Reach Remote Areas”) in 23 high malaria-endemic districts that targeted approximately 1 million people in 7,000 villages.^11^ The three cornerstones of DAMaN are: 1) Mass Screening and Treatment (MSAT) during “Malaria Camps” using a bivalent RDT followed by treatment of RDT-positive cases regardless of fever status; 2) increasing use and distribution of LLINs and IRS; and 3) community education through plays and public health advertisements. Promisingly, implementation of DAMaN has coincided with a drastic reduction of malaria in Odisha.^12^ However, rigorous scientific evaluation of DAMaN and other ongoing MCP measures in the state is key to confirming the success of the approaches. Indeed, the MCP in Odisha has been vocal about the need for such scientific evaluation through partnerships with “. . . national and international agencies for operational research to understand the impeding factors better and explore ways for malaria elimination at the earliest” (Abstract 87, Dr. M. M. Pradhan, National Congress of Parasitology and Global Summit on Malaria Elimination Sept. 26–28, 2019, Jawaharlal Nehru University, New Delhi https://www.jnu.ac.in/content/scmm-organises-30th-national-congress-parasitology-global-summit-malaria-elimination). This has led to an ongoing partnership among the CSCMi, the Community Welfare Society Hospital (CWSH; a private tertiary multispecialty healthcare institution in Rourkela, Odisha), the ICMR-Regional Medical Research Center (ICMR-RMRC, in Bhubaneswar, Odisha), and the NVBDCP of Odisha state, to study the effectiveness of DAMaN. This is a prime example of a partnership between MCP policy and control stakeholders with basic science researchers and public health advocates. The ICMR-RMRC Bhubaneswar^11^ and CSCMi/CWSH^13^ efforts differ in respect to their core epidemiological study designs, operational modalities, and geographical settings of the study regions, but, thus, also complement each other. The inclusion of Dr. M. M. Pradhan, the orchestrator behind the DAMaN initiative and currently serving as Additional Director of Public Health Services in the Odisha Department of Health & Family Welfare, and former Program Officer of the Odisha state NVBDCP, as a central member of both the CSCMi/CWSH and ICMR-RMRC Bhubaneswar initiatives ties the collaborations together and bridges the gaps between multidisciplinary scientific research and state-sponsored health-implementation programs.

Results from the CSCMi phase 1 cluster-assigned quasi-experimental study in 15 villages of Keonjhar and Jharsuguda districts suggest that the DAMaN intervention is associated with reduced malaria in the study villages and, thus, is a promising, financially feasible approach for malaria control in rural settings (Ompad, J., manuscript in preparation). A much larger, phase 2 cluster randomized trial of 80 villages in six districts is underway. Thus, the CSCMi is having a direct impact on malaria policy and public health programs in Odisha. At the district levels, for example, in Keonjhar, a populous region (∼1.8 million) inhabited mostly by tribal communities in forest fringes and mining areas, affected by multiple vector-borne diseases, with limited resources, infrastructure, and manpower, it could be extremely challenging to implement well-planned public health initiatives. According to Odisha Government officials and senior district health administrators, for example, Dr. D. Das, the Additional District Public Health Officer and District Malaria Officer of Keonjhar, who leads the vector-borne disease operations in the district, interactions with the CSCMi are satisfying the need for scientific evaluation of new health-implementation programs like DAMaN there, as well as providing capacity building and training of existing healthcare staff with new technologies. Furthermore, the collaboration with an international team is producing a much-needed boost to encourage research through organization of district-level meetings and workshops, as well as an increased social awareness of malaria and other vector-borne disease research. At the state level, the results from these two effectiveness trials will provide the scientific basis for the Odisha State MCP program to revise, redirect, and refocus DAMaN at the national level, and may also have wide-reaching consequences for MCPs in other endemic regions of the globe.

### Developing research infrastructure in private healthcare institutions.

A widely acknowledged yet relatively unexplored area by state-sponsored malaria control and elimination programs is the role of private healthcare institutions and clinics situated in malaria-endemic regions.^14^ These remain an underutilized resource that could serve as sentinel sites for malaria surveillance. Engagement of private healthcare institutions for malaria elimination programs requires devising uniform policies and platforms, not only for malaria case detection, reporting, and data sharing, but also for training, guidance, and monitoring of malaria diagnostic standards, case management strategies, and adherence to treatment guidelines.^15^^,^^16^ The CSCMi has built capacity and developed infrastructure at several private hospitals including the Steel Authority of India Ltd., IGH in Rourkela, Odisha, a public sector undertaking (PSU) tertiary hospital in Odisha undertaking seminal research in cerebral malaria (CM) pathogenesis as part of the CSCMi;^17^^–^^19^ and at the private multispecialty CWSH in Rourkela, which is leading the DAMaN effectiveness trial described previously. Despite the huge potential and value of incorporating private healthcare institution data, obstacles remain, such as the identification of the private clinics and accessibility of the data.^14^

### Data and information sharing.

**Figure 1.**
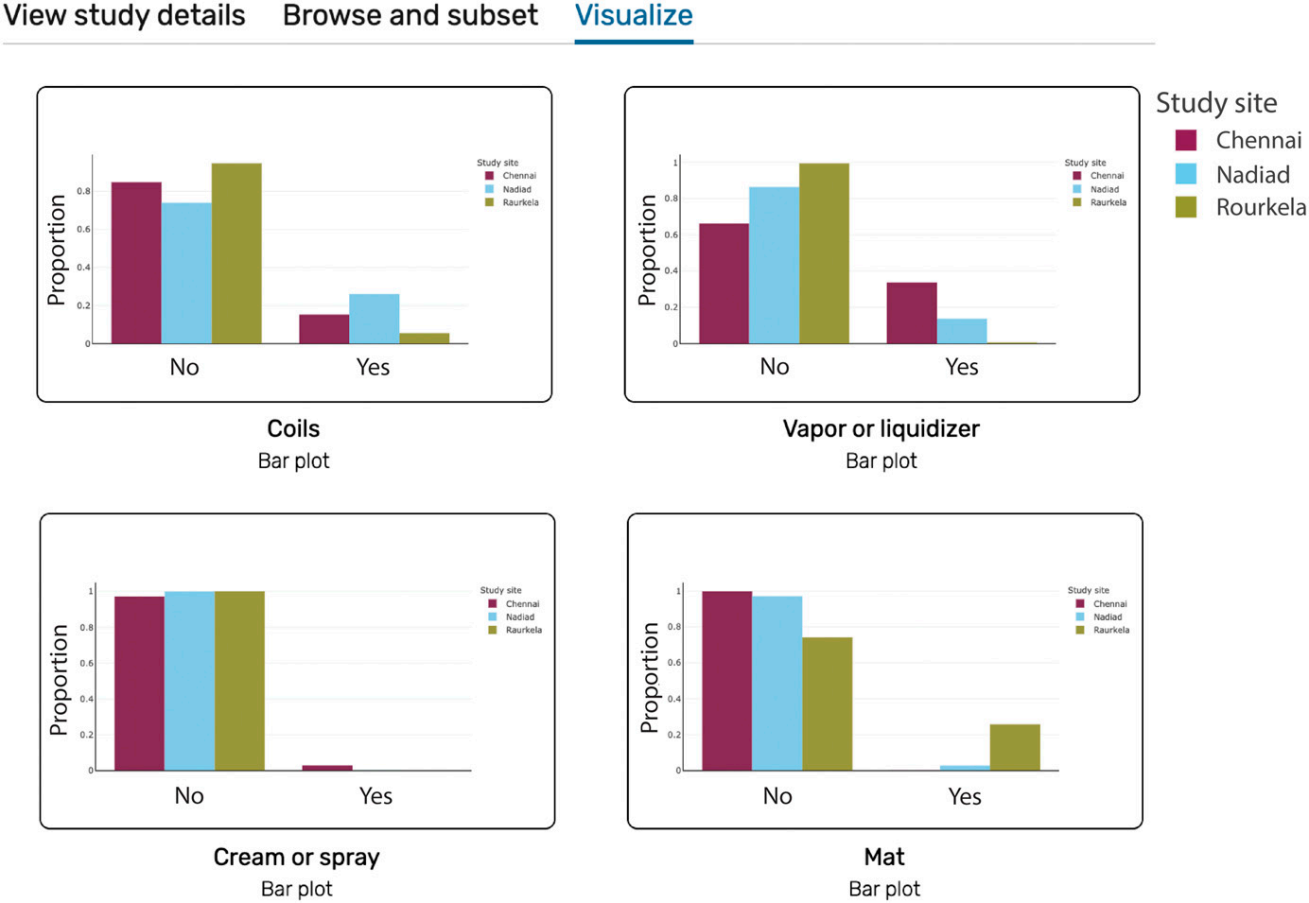
Screenshot from ClinEpiDB showing plots looking at the proportional use of personal mosquito repellents such as coils, vapor or liquidizer, cream or spray, and mats stratified by CSCMi study site. The study site Chennai is in maroon, Nadiad is in light blue, and Rourkela is in pea green. These plots can be accessed and modified in real-time directly through the ClinEpiDB website (https://beta.clinepidb.org/ce.beta/app/workspace/analyses/DS_a5c969d5fa/3c7df7a/import).

Providing public access to data collected as part of the studies of the CSCMi is an important element of the Center. Data sets have been made publicly accessible via ClinEpiDB.org, PlasmoDB.org, and VectorBase.org^20^^,^^21^ (Table 1). These open-access resources offer more than just a data repository by standardizing data workflows and structures and offering powerful analytical tools that work in an Internet browser. This standardization of data in turn supports data-mining and querying across datasets, which lets researchers, public health officials, and others ask more advanced questions, and increases the impact of our data. For instance, an immunologist interested in designing a vaccine against *P. vivax* could start by looking at our serology data and expand their query to include *P. vivax* antibody data from Peru as well. In VectorBase, a public health official could confirm whether the abundance of different malaria vectors is changing in Odisha, or look for the presence of other mosquitos, like Dengue vectors, in our data. In ClinEpiDB, the mapping of variables to ontology terms makes it easier to search across all six of our studies of various designs—including longitudinal cohort, cross-sectional, and surveillance studies—for malaria risk factors, to compare diagnostic tests, and more. Figure 1 shows how a public health official interested in the use of mosquito repellents could create a series of plots looking at the reported use of personal mosquito repellents across study sites in the India ICEMR cross-sectional study^22^ using the data visualization tools available in ClinEpiDB. The plots show low overall use of personal mosquito repellents, but substantial differences in the most common repellent form by geographic location. Thus, extending public access to all stakeholders and facilitating their interrogation of the data via platforms like ClinEpiDB increases the utility and impact of data collected.

**Table 1:** Datasets that have been integrated into a data resource, with a link to the dataset and brief description

Study name	Data resource	Data ID and link	Data description and reference
India ICEMR Cross-sectional	ClinEpiDB	DS_a5c969d5fa	Cross-sectional epidemiological data at 3 sites 2012–2014^37^
	PlasmoDB	DS_4267c95a1c	Human serum antibody levels^38^
India ICEMR Cohort	ClinEpiDB	DS_05ea525fd3	Longitudinal epidemiological data at 2 sites 2013–2015^22^
India ICEMR Fever Surveillance	ClinEpiDB	DS_4902d9b7ec	Data from a fever clinic 2016–2017^39^
India ICEMR Severe *P. vivax* and *P. falciparum* Cohort	ClinEpiDB	DS_cbd9087ebc	Severe malaria cohort 2016–2017^40^
India ICEMR Behavior Cross-sectional	ClinEpiDB	DS_21205ebb16	Cross-sectional epidemiological data at 1 site 2017^41^
India ICEMR Meghalaya Cross-sectional	ClinEpiDB	DS_4670e06911	Cross-sectional epidemiological data 2018–2019^23^
India ICEMR DAMaN Quasi-experimental Stepped-wedge	ClinEpiDB	DS_206a27bc7c	Phase 1 of a DAMaN Malaria Camp effectiveness trial 2019–2021^13^
Entomology	VectorBase	VBP0000162	Mosquito abundance and host blood meal data 2013–2014^42^
		VBP0000182	Larval abundance in water samples 2013–2014^5^
*Plasmodium* whole genome sequences	PlasmoDB	DS_6a99014266	*P. vivax* and *P. falciparum* genomes^24^
		DS_079607ca4d	*P. vivax* genome diversity and hybrid selection project^25^

## EMERGING NEEDS AND NEW RESEARCH QUESTIONS FROM 12 YEARS OF THE CSCMI

Given the impact of the India ICEMR on malaria policy and public health described previously, and the research results described in the accompanying manuscript,^2^ what are the emerging needs and new research questions that have been identified as CSCMi activities have progressed?

### The continued importance of vector and transmission research.

The vector and transmission studies undertaken by the CSCMi have proven valuable to MCP efforts, and continued surveillance will be important to track changes in mosquito prevalence, identify new putative vectors, and track insecticide resistance. For example, our studies in the Northeastern State of Meghalaya identified new mosquito species (*Anopheles xui* and *Anopheles dissidens*) that were not previously reported in India, and identified others which have been shown to be potential vectors in many Southeast Asian countries.^23^ We recognize the need to involve government stakeholders beyond the NVBDCP in policy and control decisions, in particular to include agricultural institutes and rice authorities because many known malaria vectors breed in rice fields and associated irrigation channels. And our assessment of the quality of morphological identification of *Anopheles* being undertaken by NVBDCP entomologists using molecular identification as a comparator found ∼50% of the samples had been misidentified. Thus, continued capacity building and training in vector biology is also required to meet the challenge of mosquito biodiversity in the region.

### The age of multiomics data generation.

Our CSCMi studies produced the first *P. falciparum* and *P. vivax* whole genome sequences from India,^24^ and led to the finding that *P. vivax* has greater genetic diversity than *P. falciparum*, with significant consequences for control and elimination strategies of these two species. Our subsequent population genomics study analyzed ∼180 *P. vivax* genomes from 11 countries including India, and identified signals of natural selection that suggest that *P. vivax* is adapting to regional differences in the human host and *Anopheles* vector.^25^ In short, *P. vivax* in one endemic region looks genetically very different from *P. vivax* in another. This diversity requires detailed sampling so that the vivax research community can have access to high-quality genomes of today’s circulating strains. In addition, *P. vivax* subtelomeric regions contain important immune evasion gene families^26^ and their characterization is hindered by the absence of high-quality contiguous subtelomeric sequences of circulating strains. Our ongoing studies aim to redress this imbalance through generating high-quality telomere-to-telomere (T2T) reference genomes, plus their transcriptomes, metabolomes, and 3D genome organization^27^ from *P. vivax* isolates collected at several locations in India. A lack of ’omics reference resources is also apparent for other species of *Plasmodium* parasite and their *Anopheles* vectors in India. A partnership between Indian genomics scientists and CSCMi researchers to address this lack could accelerate the development of a multipurpose omics resource for malaria basic research.

### Severe and cerebral malaria: no time to reduce the pace of research.

The malaria landscape has undergone significant changes over the last 12 years, leading to the emergence of new challenges locally and globally. We must adapt our approaches accordingly as follows:

#### The need for multicentered studies.

Our neuroimaging work in Rourkela has shown that the pathogenic mechanisms leading to fatal CM differ between adults and children.^18^ However, we recently compared blood and MRI profiles between our cohort and a Malawian one with different age groups, and identified common brain swelling determinants at both sites.^17^ As our understanding of the pathogenesis of CM widens, it has become increasingly evident that findings must be compared among different sites, endemicities, and age groups to rigorously design effective adjunct therapies. These have been unsuccessful so far,^28^ and in view of our recent findings, a silver bullet will be unlikely to work across the malarious world.

#### Focus on “control” patients.

Our team demonstrated that patients matching the WHO definition for uncomplicated malaria (UM), despite being sick enough to be hospitalized, frequently show brain changes identifiable through magnetic resonance imaging (MRI).^18^ These patients have higher parasite loads than observed in UM cases from Malawi,^17^ suggesting that there is a spectrum of morbidity in this group. Further investigations are needed in UM patients to better understand the potential resultant sequelae and/or long-term effects on coinfection, comorbidities, and mortality, which have all been described in “asymptomatic” malaria.^29^

#### The shifting definition of CM.

Our neuroimaging analyses indicate that brain involvement in severe falciparum malaria is frequent, and that coma defined by a Glasgow Coma Score of 11 or below does not allow capturing all patients with neurological complications.^30^ These findings challenge the current clinical definition of CM, and since neuroimaging facilities are seldom available in endemic areas, new tools, such as biomarkers associated with brain changes on MRI, are needed to identify patients with silent CM.^31^ In addition, neurological sequelae in severe non-CM (SNCM) patients have never been assessed but their MRI patterns combined to high plasma levels of S100B, a marker of brain injury, suggest the likelihood of subtle but long-term issues. New strategies designed for treatment and follow-up might, therefore, be needed to support recovery and rehabilitation. Recently published data indicate the occurrence of similar pathogenic pathways between CM and Alzheimer’s disease (AD).^32^ We are leveraging the unique CSCMi repository of plasma and MRI datasets to evaluate whether pathogenic events such as axonal injury and microglia dysfunction are involved in both AD and CM.

#### Importance of other clinical parameters in severe malaria.

Although evidence is mounting for the pivotal role of platelets in brain swelling during CM,^17^^,^^33^ other clinical parameters such as retinopathies did not correlate with disease severity in our cohort.^34^ Given the wide spectrum of brain changes we identified across CM, uncomplicated, and SNCM patients using neuroimaging, additional correlation studies are needed to investigate the association of: 1) retinopathies, a diagnostic sign in pediatric CM;^35^ 2) *P. falciparum* binding variants; and 3) other hematological parameters with specific MRI patterns in severe malaria. These projects are all currently ongoing as part of the CSCMi.

### An emerging need: health systems strengthening.

Widespread distribution of LLINs in endemic areas has contributed to the dramatic reduction in malaria incidence in the Northeastern state of Meghalaya in India. In policy circles, there is a tendency to attribute the change to LLINs alone. But analysis of the decadal data indicates that although LLINs potentially contributed, a fall in malaria incidence had already begun.^36^ Some of the factors involved health system strengthening efforts over the past 10 years, for example innovations such as the “Tura model” of test and treat at the village-level potentially helped (https://theshillongtimes.com/2021/11/03/malaria-related-morbidity-and-mortality-rates-plummet-in-state/). Thus, empowering healthcare systems at the grassroots level, including addressing public health issues by focusing not only on curative aspects but more on preventive as well as enabling aspects, is an emerging need.

## CONCLUSIONS AND CLOSING THE GAPS

Over 12 years, the CSCMi has undertaken innovative and pioneering research leading to the publication of 10s of open-source papers, and generation of terabytes of epidemiological, genomics and vector data deposited into public databases for use by malaria researchers. Other achievements include: 1) human capacity building through international conferences and workshops in topics as diverse as *Anopheles* morphological identification to next generation sequencing for the training and career development of Indian technicians, PhD students, postdoctoral fellows, and junior faculty; 2) transfer of novel technologies such as hand-held genomic sequencing devices and microEEG devices; 3) infrastructure development through renovation and construction of new laboratories and clinics; and 4) development of interinstitutional collaborations within India. Although there has been some shaping of malaria policies and public health approaches, gaps between research and policy continue to exist. We plan to develop mechanisms to identify and address these gaps, an especially important goal due to the changing malaria prevalence that has been apparent in India over the past 12 years.
